# More Similarity if Different, More Difference if Similar: Assimilation, Colorblindness, Multiculturalism, Polyculturalism, and Generalized and Specific Negative Intergroup Bias

**DOI:** 10.5964/ejop.3715

**Published:** 2022-11-30

**Authors:** Anastasia Batkhina, John W. Berry, Tomas Jurcik, Dmitrii Dubrov, Dmitry Grigoryev

**Affiliations:** 1 Center for Sociocultural Research, HSE University, Moscow, Russian Federation; 2 Department of Psychology, Queen's University, Kingston, Canada; 3 Department of Psychology, HSE University, Moscow, Russian Federation; Heriot-Watt University, Edinburgh, United Kingdom

**Keywords:** diversity ideologies, assimilation, colorblindness, multiculturalism, polyculturalism, intergroup bias

## Abstract

The creation of a social climate where all ethnic groups can harmoniously coexist is a central challenge for many countries today. Should we emphasize similarities and common ground or, conversely, recognize that there are important differences between groups? The current study examined relations between diversity ideologies (assimilation, colorblindness, multiculturalism, polyculturalism) and generalized and specific intergroup bias (against Chechens, Belarusians, Uzbeks, Chinese, and Jews and Muslims) among ethnic Russians (N = 701). In Study 1, colorblindness (ignoring differences) and polyculturalism (emphasizing interconnectivity) were associated with lower generalized intergroup bias and lower bias against Chechens, Uzbeks, and Chinese, but not Belarusians. Bias against Belarusians was lower among those who endorsed multiculturalism (emphasizing differences). In Study 2, multiculturalism was associated with higher implicit bias when the target was a Chechen but in general more proximal variables (positive or negative contact experience and perceived group similarity) were more robust predictors of intergroup bias than diversity ideologies. In Study 3, colorblindness and polyculturalism were related to lower levels of fearful attitudes against Muslims. Colorblindness was also associated with lower levels of Antisemitism in contrast to multiculturalism that had an opposite association. We place these results in the context of cultural distance and existing cultural stereotypes about different groups among the majority of Russians. The strengths and weaknesses of each diversity ideology for the mainstream cultural group are discussed. The results of the current study suggest that the most fruitful strategy for mainstream cultural groups for maintaining harmonious intergroup relations in diverse societies might be that of optimal distinctiveness.

In recent years, the study of intergroup relations has become paramount given the urgent need to manage the growing cultural and ethnic diversity of societies. The creation of a social climate where all ethnic groups can coexist harmoniously is one of the main challenges for many countries today (Berry, 2017). Multicultural policy is one of the ways to cope with this challenge in pluralistic societies (Berry & Ward, 2016). However, in the current political discourse, multiculturalism is associated with globalization and is strongly criticized by populist movements around the world (see e.g., Berlet, 2011). Multicultural policy was developed in the 1970s in certain Western societies (e.g., Canada and Australia) as a means to cope with the growing cultural and ethnic diversity, and the participation and inclusion of such groups in the larger society^1^1The first public policy to explicitly seek to manage interethnic relations in a plural society was proposed in 1971 by the Federal Government of Canada. This policy of multiculturalism was intended to achieve interethnic harmony by adopting two principles and programmes: to promote the extant cultural diversity as a way of providing everyone a secure sense of place in the society; and to promote social contact and interaction among groups. It is clear that this policy is not just about diversity, but also about participation; together these two components of the policy sought to achieve mutual interethnic acceptance. Conceptual analyses of the policy by Berry (1984) and Kymlicka (2016) confirmed that these two emphases formed the core of the policy. However, in the present paper, we adopt (but do not accept as correct) the common usage of the concept of multiculturalism as emphasizing only the first aspect (diversity).. It was seen as an alternative to the previously widespread policies of assimilation and segregation (Guimond et al., 2014).

An extensive socio-psychological analysis of the Canadian policy of multiculturalism was proposed in a multi-component model covering cultural, social, and communication levels of intercultural relations (Berry, 1984, 2006). This model postulates three hypotheses based on the policy: the multiculturalism hypothesis (having cultural, economic and personal security serves as a basis for the acceptance of ‘others’ in a plural society); the integration hypothesis (having multiple cultural identities promotes personal wellbeing and tolerance of others); and the contact hypothesis (engagement with others promotes acceptance of others). Recently, Berry et al. (2022) carried out an evaluation of these hypotheses in 21 societies and found supporting results (see also Berry, 2017; Inguglia et al., 2020). This line of thinking and research considers both cultural maintenance and social participation to be of equal importance in achieving intercultural harmony.

At much the same time, there has been a parallel stream of research that focuses on issues of maintenance of cultural diversity, but omits considering issues of the participation and inclusion of these diverse groups in the life of the larger national society (Grigoryev & Berry, 2021). The main issue addressed in these studies is: should we emphasize similarities and common ground or, conversely, recognize that there are important differences between groups? This question implicitly recognizes various diversity ideologies, which include both a socially constructed superstructure (which creates and transmits social ideas about the organization of a particular plural society) and a motivational substructure. These ideologies generally constitute a common belief system about how a plural society should function; in short, they provide a cognitive-motivational framework for the interpretation and expected accommodation to the social environment (Badea, 2017). Such recognition can be achieved in several ways, if supported by relevant social norms (Guimond et al., 2013).

The existing evidence for the degree of benefit for these differing views is mixed and inconsistent (see e.g., Whitley & Webster, 2019, and brief review below). The current study aims to examine whether these inconsistencies could be explained by taking into account the specific outgroup being studied, and whether explicit or implicit bias is used as an outcome measure. In a series of studies, we examine how the most commonly studied views on diversity associate with both explicit and implicit bias against various outgroups, chosen to represent different degrees of cultural similarity to the majority group. This paper contributes to the literature by comparing various diversity strategies that can foster the maintenance of harmonious intergroup relations in diverse societies from the perspective of a mainstream cultural group (Russians). We explore the role of cultural similarity between groups, and the role of optimal distinctiveness. Individuals follow two fundamental and competing human needs: the need for inclusion and the need for differentiation, which can be met by membership in moderately inclusive (optimally distinct) groups (see Leonardelli et al., 2010). Thus, in line with previous research, we address the relevance of a group-specific approach to intergroup attitudes, by taking into account the specific cultural context.

## Review of Diversity Ideologies

Since many countries have set out to cope with their growing cultural and ethnic diversity, the advantages and disadvantages of specific interethnic policies and diversity ideologies are actively discussed in the social science literature (e.g., Correll et al., 2008; Gagnon & Iacovino, 2016; Guimond et al., 2014; Levin et al., 2012; Moghaddam, 2012; Ng Tseung-Wong & Verkuyten, 2018; Pedersen et al., 2015; Rattan & Ambady, 2013; Rosenthal & Levy, 2012; Scott & Safdar, 2017). The four most frequently considered ideologies of intergroup relations in the aspect of view on diversity are: (1) *assimilation* promotes the existence of one common cultural group in society, where ethnic minorities and migrants are expected to adopt the mainstream culture while completely rejecting their own; (2) *colorblindness* assumes that relations between groups can be improved by ignoring intergroup differences: there are no groups, there are only different personalities; (3) *multiculturalism* recognizes the differences between ethnic groups and promotes the maintenance of this diversity, recognizing the importance of group membership;^2^2For example, a framework to define four ways of interacting was proposed by Berry (1974). The concept of integration combined both maintenance of culture, and of participation. Assimilation was defined as not maintaining culture while seeking to fully participate. Separation was the concept used to represent cultural maintenance without seeking participation. Marginalization was the concept used to refer to neither maintenance nor participation. The first scale to assess these two elements was by Berry et al. (1977), called Multicultural Ideology. This scale had items that placed the acceptance of integration (i.e., both cultural maintenance and equitable participation), at one end, and items that valued assimilation, segregation and marginalization at the other end. Empirical examinations of this scale support that it is a unidimensional concept and scale (Berry et al., 1977; Berry & Kalin, 1995). (4) *polyculturalism* implies a close relationship between all ethnic groups living in the same society, and pays less importance to the boundaries between them; in other words, all cultures are not isolated systems but are the product of intergroup interaction; recognition of group membership is necessary, but the emphasis is shifted to the interconnections between groups (for review, see Guimond et al., 2014; Pedersen et al., 2015; Rattan & Ambady, 2013; Rosenthal & Levy, 2012). We summarize these different diversity ideologies in Table 1.

**Table 1:** Taxonomy of Diversity Ideologies

	Diversity ideologies		
Rejection/Ignoring of cultural diversity		Acceptance of cultural diversity	
*Assimilation*	*Colorblindness*	*Multiculturalism*	*Polyculturalism*
One group with a common mainstream culture; eliminating minority group memberships	No groups, only unique individuals; ignoring group memberships	Plurality of different groups; acknowledging and valuing group memberships and differences between groups	Plurality of interconnected groups; acknowledging group memberships by valuing interconnection between groups

The concept of *polyculturalism* is the only one of the four that is not based on assumptions underlying the intergroup contact model (i.e., polyculturalism is not considered as a condition for obtaining a beneficial effect from subsequent intergroup contact). It assumes that ethnocultural groups around the world have always influenced and continue to influence each other, rather than forming a common identity and common goals with other groups (Rosenthal & Levy, 2012).

In the social psychology literature *assimilation* is based on the principle of recategorization within a common ingroup identity model and the similarity-attraction paradigm; *colorblindness* is based on the principle of decategorization and the model of personalization; *multiculturalism* is based on principles of categorization and the mutual intergroup differentiation model; together, they form three different paths to positive intergroup contact (Guimond et al., 2014). There are studies that show that learning multiculturalism (multicultural priming) can enhance intergroup differentiation and reinforce stereotypes (Hodson & Dhont, 2015; Morrison et al., 2010). Also, a few empirical studies have shown, for example, that polyculturalism, unlike multiculturalism, is not associated with essentialism (Bernardo et al., 2016; Wilton et al., 2019), but at the same time is associated with a greater interest in diversity and comfort of living in a diverse ethnic and cultural environment (Rosenthal & Levy, 2012).

We recognize, however, that intergroup contact may not necessarily be associated with beneficial adaptation outcomes at the community level, given that numerous studies have shown poor or mixed outcomes with respect to cohesion and well-being in areas associated with greater ethnocultural diversity (e.g., Putnam, 2007; van der Meer & Tolsma, 2014; see also Paluck et al., 2019). However, in the national surveys in Canada, in analyses at the level of neighborhoods was found that with the proportion of a particular group being greater, the attitudes toward that group by non-members were more positive (Berry, 2006). Also, some negative effects, according to the latest findings, are typical for the short term and may be mitigated with time (see Ramos et al., 2019). An 18-year longitudinal study in the UK suggests that negative effects for cohesion may depend on whether people choose to stay or move from their diversifying neighborhoods, with 'stayers' showing negative effects while 'movers' to more *homogeneous* neighborhoods reporting improved attitudes (Laurence & Bentley, 2016). It is possible that cultural distance between the mainstream and ethnocultural groups may moderate some of these links between diversity and outcomes, as greater cultural distance has been associated with problems in adjustment and social adaptation (see Galchenko & van de Vijver, 2007). Clearly, the topic of diversity remains controversial. However, the purpose of this study is on ideologies and intergroup bias in the majority group rather than community level outcomes associated with diversity itself. We thus turn to some of the conceptual, empirical, methodological and contextual variability associated with diversity ideologies.

As Grigoryev et al. (2020) mentioned, it is important to understand what is meant by multiculturalism (conceptualization) in the literature and how it is measured (operationalization). For instance, some measures were shown to have only about 27% common variance (see Rosenthal & Levy, 2012). Different conceptualizations, operationalizations, and levels of analysis can complicate the interpretation of findings. Thus, in the literature there are contradictory results about the role of each of the diversity ideologies in pathways to positive intergroup relations (Pedersen et al., 2015; Rattan & Ambady, 2013).

For example, a recent meta-analysis (Whitley & Webster, 2019) found that explicit prejudice was positively related to assimilation but negatively to multiculturalism and colorblindness. Compared to colorblindness, this association was stronger for multiculturalism, which was also found to be associated with less implicit prejudice. These contrasting findings may also be due to different contexts since the historically established forms of interethnic interactions vary between each setting (Ng Tseung-Wong & Verkuyten, 2018). Further, the findings varied with methodology and country. In this regard, Pedersen et al. (2015) argued that more research is needed to understand and recognize the mechanisms and consequences of diversity ideologies. Our research follows this admonition, and extends the examination of these ideologies to a unique population, and employs new object groups. In the present study, we use the conceptualizations and operationalizations of diversity ideologies proposed by Rosenthal and Levy (2012) that only concern with views on diversity, excluding issues of the participation and inclusion of diverse groups (e.g., in contrast Berry, 2017).

## The Context of the Study: Russia

We turned to the Russian population to study diversity ideologies, given that this population is highly diverse and has been understudied (e.g., Bessudnov, 2016; Jurcik et al., 2013). The study of diversity ideologies is especially important in this context, since the Russian population comprises more than 190 ethnic groups and the United Nations estimated the Russian Federation as the world's second-leading country in the number of immigrants for 2013. There are several groups of immigrants in Russia (e.g., Ukrainians, Uzbeks, Tajiks, Azerbaijanis, Moldovans, Kazakhs, Armenians, Belarusians, Chinese, and others) which have various cultural distances from Russians, and have a different tendency to communicate with the host population. For example, Russians tend to categorize immigrants from Transcaucasia and Central Asia into a common outgroup with internal migrants from Russian regions of the North Caucasus, in contrast to immigrants from Ukraine or Belarus (Grigoryev et al., 2018b, 2019). Historically established ethnic hierarchies remain typical for post-Soviet Russia (Hagendoorn et al.,1998). Attitudes towards immigrant groups are likely to differ depending on the specific group, and thus studying broad attitudes towards immigrants in general will obfuscate such variance (Satherley & Sibley, 2016).

We addressed bias against four ethnic groups which have different cultural distances from the Russian mainstream population and vary on the ethnic hierarchy and in stereotypes: Chechens, Belarusians, Uzbeks, and Chinese. Chechens are a Caucasian ethnic minority living in North Caucasus region of Russia, predominantly Muslims, a largely stigmatized group, and often represent the image of a typical internal migrant. Belarusians are an East Slavic ethnic group living in the former-Soviet republic of Belarus, are predominantly Orthodox Christian, have a large diaspora in Russia, are typically very fluent in Russian and have a culture that is very close to European Russians. Uzbeks are a Turkic ethnic group living in the former-Soviet republic Uzbekistan, predominantly Muslims, have a large diaspora in Russia, and often represent the image of a typical immigrant worker from a post-Soviet republic in Russia. Of the four groups, the Chinese are the only group that never lived with Russians within the framework of one state, and have the largest cultural distance from Russians, and often represent the image of a typical immigrant worker from far abroad in Russia (Grigoryev et al., 2019). Thus, including bias against these very different ethnic groups in our study, we ensured overlapping common variation of generalized intergroup bias against ethnic groups while providing richer information that would allow us to compare the diversity ideologies towards these disparate groups. Additionally, we considered special forms of intergroup bias: anti-Semitism and negative attitudes towards Muslims and Islam. Hence, our research covered both explicit and implicit intergroup bias and different levels of generalization.

## Overview

In Study 1, we examined the model of relationships between assimilation, colorblindness, multiculturalism, polyculturalism and generalized and specific intergroup bias. In order to take into account the influence of the specific context of intergroup relations, we added to our model a latent factor that reflected contact experience potentially associated with intergroup bias and tension according to the literature, including frequency and positivity of interethnic contacts (e.g., Pettigrew & Tropp, 2006; Visintin et al., 2017a), positive interethnic emotions (e.g., Seger et al., 2017; Visintin et al., 2017b), and perceived neighbourhood ethnic density (e.g., Jurcik et al., 2015). We composed latent factors to reflect intergroup bias against each of the considered groups using measures of willingness for intergroup contact and endorsement of discrimination of immigrants in the socioeconomic domain as manifest variables; the willingness for intergroup contact and endorsement of discrimination of immigrants in the socioeconomic domain are both informative aspects of intergroup bias since these variables greatly reflect common dealings with ethnic outgroups (Grigoryev et al., 2018a). Also, based on the assumption that the bias against one specific outgroup is, for the most part, associated with bias against other outgroups (Hodson et al., 2017; Zick et al., 2008), we added a common second-order latent factor to generalize the variation of intergroup bias against specific groups. Moreover, the outgroup homogeneity effect can also be tested by including the generalized intergroup bias as a target outcome (see Musso et al., 2017). For example, Montreuil and Bourhis (2001) argued that it is possible that majority group members may not endorse distinctive acculturation orientations toward different immigrant target groups but instead construct ‘generic’ acculturation orientations, viewing all immigrants as members of one homogeneous outgroup. They noted that studies documenting the outgroup homogeneity effect have shown that majority group members have a tendency to perceive less variability in the traits describing minority outgroup members than those describing ingroup members.

Further, in two subsequent studies, we attempted to conceptually reproduce and expand the findings of Study 1. In Study 2, we added implicit bias as the main outcome variable. Some have argued that implicit bias (i.e., bias based on associations without our conscious knowledge) is essential for taking into account individual differences in expressing intergroup attitudes and predicting other outcomes; for instance, the Implicit Associative Test (IAT) beyond self-report methods can be useful in assessing implicit bias (Greenwald et al., 2003; Hewstone et al., 2002). We excluded Uzbeks from this part of the research to reduce the cognitive burden on participants when responding to the IAT as Uzbeks and Chechens are from the same quadrant of stereotypes (see Grigoryev et al., 2019) and in Study 1 (see results below) the association between diversity ideologies and bias against these groups was similar. Moreover, we included additional covariates (perceived similarity and contact experience). Finally, in Study 3, we examined the association between diversity ideologies and two forms of intergroup bias: anti-Semitism and fear of Muslims. The decision to focus on fear of Muslims was motivated by the findings of Study 1, where we observed a similar pattern in the associations between diversity ideologies and bias against Chechens and Uzbeks who are predominantly Muslims. Here we examined whether this pattern would hold with the general attitude towards Muslims. To add a comparative perspective, we additionally measured anti-Semitism, to contrast the visible cultural minority of Muslims with a less visible, more integrated group of Jews. We also included ethnic and national identification as additional covariates (e.g., Levin et al., 2012; Morrison et al., 2010; Ng Tseung-Wong & Verkuyten, 2018).

## Hypotheses

Although there is little research in the Russian context, we expected the results would be consistent with the following patterns:

1. Based on the evidence (e.g., Guimond et al., 2013; Pedersen et al., 2015; Perry et al., 2015), we expected that assimilation would be positively associated with biases against all groups.
2. In view of previous studies (e.g., Levin et al., 2012), and post-Soviet attempts to construct a common civic identity in Russia focused on identification with society, state, and country (Codagnone & Filippov, 2000), we expected that colorblindness would be negatively associated with all group bias.
3. Taking into account the benefits of multiculturalism for intergroup attitudes (Whitley & Webster, 2019), we posited that multiculturalism would be negatively associated with generalized bias. However, since multiculturalism is sensitive to threat from external sources, and perceiving greater threat from immigrant groups was associated with lower endorsement of multiculturalism (Rattan & Ambady, 2013), we expected it to be more likely that multiculturalism would be negatively associated with bias against Belarusians than bias against other (more culturally distant) groups. Alternatively, there are differences in findings between groups more traditionally associated with diversity in the country compared to those that are not (e.g., in the US multiculturalism is associated with positive attitudes towards African Americans, Latino Americans, and Asian Americans, but not towards Arab Americans; Rattan & Ambady, 2013). Hence, multiculturalism may also be negatively associated with intergroup bias against all groups except the Chinese in our Russian sample.
4. Polyculturalism relates to various positive intergroup attitudes more consistently than multiculturalism (Pedersen et al., 2015; Rosenthal & Levy, 2012); it is thus more likely that polyculturalism is negatively associated with all group bias.

## Study 1

In our first study we examined generalized and specific intergroup bias in relation to assimilation, colorblindness, multiculturalism, and polyculturalism.

### Method

#### Participants

The total sample of 359 Russians from the Central Federal District of Russia included 167 women (46.5%) and 192 men (53.5%), aged from 16 to 68 years (*M* = 33.9, *SD* = 11.9); 22% participants were students; 43% were Russian Orthodox Christians; almost half of the participants had an income of about 16,250 rubles (≈ $220) per person.

#### Procedure

The data were collected online in 2017 via social media. We recruited participants using targeted, paid targeted advertisements in the most popular social network in Russia, named *VK* (a platform similar to Facebook). Participants were given a questionnaire and asked to read the instructions, which included information about the main topics discussed in the study, the confidentiality policy, and how to contact the researchers supervising the project. The informed consent of the participants was implied through survey completion. This procedure was in line with university and national Russian regulations, and thus no ethics clearance was required for this type of survey research (i.e., as it does not include medical data).

#### Measures

All measures used a 9-point Likert scale. Measures were scored such that higher scores indicated stronger endorsement of the concept.

##### Antecedent Variables

**Diversity Ideologies:** Three diversity ideologies, colorblindness, multiculturalism, and polyculturalism were assessed with 5 items (Rosenthal & Levy, 2012)^3^3All measures in this research which did not have a Russian translation were adapted by back-translation and cognitive interviews with the think-aloud technique (Willis, 2004) or took from Grigoryev and van de Vijver (2018) and Grigoryev et al. (2018b).; in addition, we developed 5 new items anchored to assess assimilation. Sample items included, "There should be no cultural differences between ethnic groups; there should be a single group and people should maintain the culture of the majority of the country's population," (assimilation, α = .75), "All human beings are individuals, and therefore race and ethnicity are not important," (colorblindness, α = .83), "There are differences between racial and ethnic groups, which are important to recognize," (multiculturalism, α = .63), and "There are many connections between different cultures" (polyculturalism, α = .78). We also included the measure of multicultural ideology by Berry and Kalin (1995) comprising 6 items to check convergent validity of these diversity ideologies measures, with sample items such as "A society that has a variety of ethnic and cultural groups is more able to tackle new problems as they occur," and "We should recognize that cultural and racial diversity is a fundamental characteristic of Russian society" (α = .88).

##### Covariate Variables

**Positive Interethnic Emotions.** Four items assessed the frequency of emotions (interest, pride, sympathy, compassion) in relation to each ethnic group (Boyle et al., 2014). A sample item was "How often were you proud of Chechens living in Russia?" (α = .90).
**Frequency of Interethnic Contacts.** We used three items to measure interethnic contact frequency (Visintin et al., 2017b). A sample item included "How many people from another ethnic group in Russia do you know personally?" (α = .85).
**Positive Interethnic Contacts.** We used five items assessing the nature of interethnic contacts for each considered group (Reimer et al., 2017). A sample item was "How often did Chinese living in Russia help you?" (α = .90).
**Ethnic Density.** We adapted a four-item measure assessing perceived neighborhood ethnic density (Jurcik et al., 2015). Ethnic density scales typically assess own ethnic group density, but in this case we measured the perceived density of *other* (i.e., non-Russian) groups (Grigoryev et al., 2018a). For example, participants were asked to think of their local area (15–20 minutes walking distance from their home) and to estimate "What proportion of all the people in this local area is of other ethnic groups?" (α = .76).

##### Outcome Variables

**Willingness for Intergroup Contact.** We used five items to assess participant openness to interact with members of each considered group: four items were included from Halperin et al. (2007) and one additional item was added about friendly relations. Sample items included, "I would agree to live in the same neighborhood with an Uzbek," and "I am willing to invite an Uzbek to a social event at my home" (for Chechens α = .92; for Belarusians α = .94; for Uzbeks α = .93; for Chinese α = .90).
**Endorsement of Discrimination of Immigrants in the Socioeconomic Domain.** We used 6 items for each considered ethnic group asking about endorsement of behaviors that reflect discrimination in the workspace, labor market, rental housing sectors, and other relevant socioeconomic domains (see Grigoryev et al., 2020; Mallender et al., 2014). Sample items included "Paying Belarusians lower wages than natives, provided equal qualifications and level of education," and "The lack of career prospects for Belarusians" (for Chechens α = .80; for Belarusians α = .62; for Uzbeks α = .86; for Chinese α = .83).

##### Additional Variables

**Marker Variable.** In order to account for method bias, we used four items measuring spiritualism from Tobacyk's revised paranormal belief scale (Lange et al., 2000). Sample items included "Your mind or soul can leave your body and travel (astral projection)," and "Reincarnation does occur" (α = .92).

#### Data Analysis

**Figure 1:**
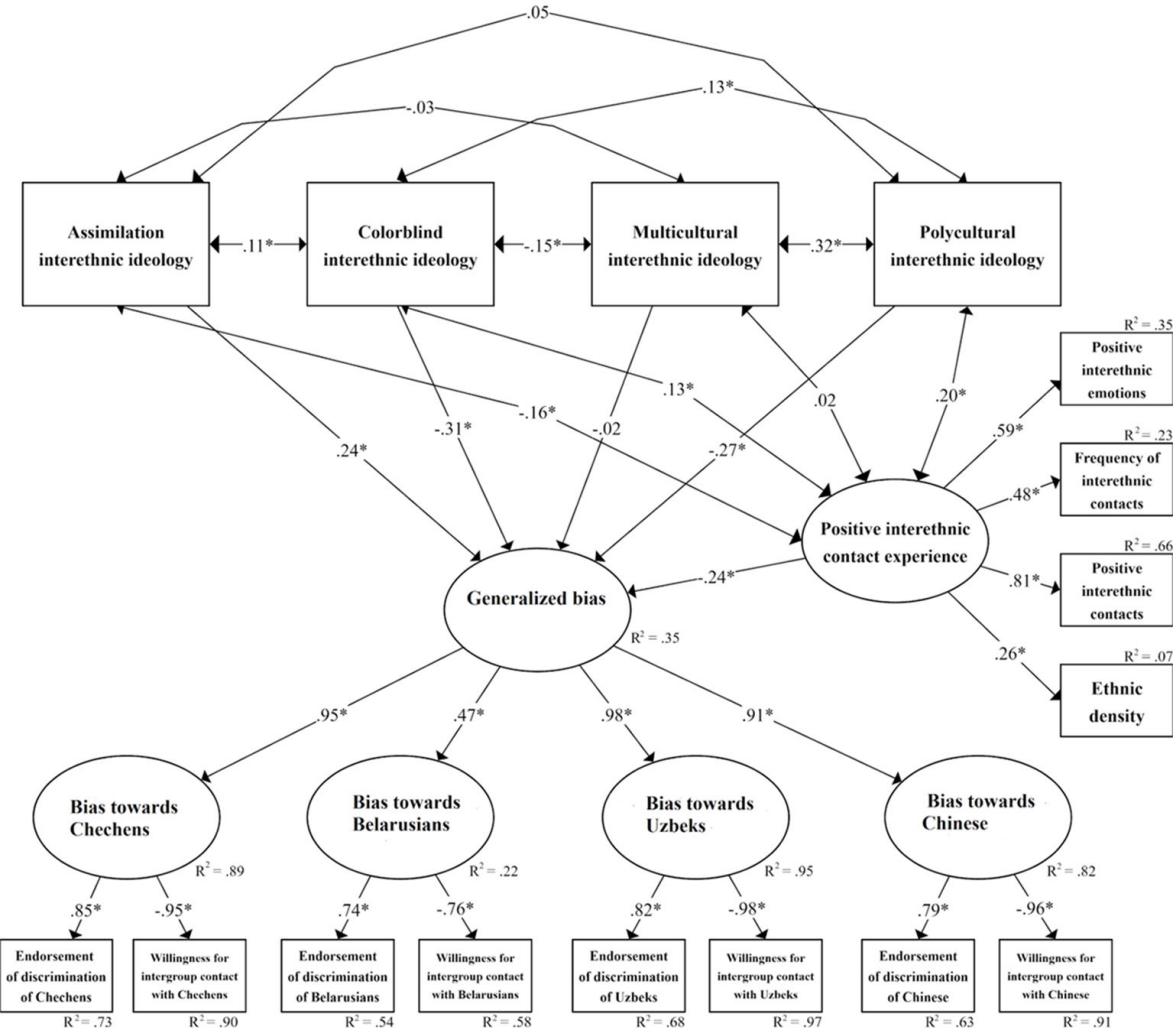
The Resulting SEM Model in Study 1 *p < .001.

Using SPSS v.24, we conducted data screening including checking for outliers and missing data. We applied partial correlation analysis with 2000 bootstrapping samples adding sociodemographic covariates (gender, age, education, income, Russian ethnicity (no/yes), affiliation to religion (no/yes), student status (no/yes), work status (unemployed/employed) as control variables to eliminate the common variation due to these differences; also, the marker (i.e., theoretically unrelated) variable was used to control for possible common method bias (e.g., response style) (see Podsakoff et al., 2003). Paired samples *t*-tests with 2000 bootstrapping samples were applied to estimate differences between the considered groups by outcome variables. Finally, we converted the partial correlation matrix to a covariance matrix and used it with the *lavaan* R package (Rosseel, 2012) to test the models applying structural equation modeling (SEM; model 1 explained generalized intergroup bias and model 2 explained specific intergroup bias, see Figure 1 below). We employed commonly recommended global fit measures: CFI > .90, RMSEA < .08, and SRMR < .08 (Kline, 2011; van de Schoot et al. 2012).

### Results

#### Preliminary Analysis

The data had no outliers or missing values. All measures had acceptable internal consistencies, with Cronbach's alpha (α) ranging from .62 to .94; the average value was .83. The measure of multicultural ideology by Berry and Kalin (1995) was positively correlated with colorblindness, *r* = .24, SE = .05, 95% CI [.13, .33], *p* < .001, multiculturalism, *r* = .18, *SE* = .06, 95% CI [.07, .28], *p* = .001, and polyculturalism, *r* = .39, *SE* = .06, 95% CI [.27, .50], *p* < .001, and negatively correlated with assimilation, *r* = -.46, *SE* = .04, 95% CI [-.54, -.37], *p* < .001^4^4These results are similar to those reported by Rosenthal and Levy (2012).. Descriptive statistics including means, standard deviations, and partial correlations are presented in Appendix A available in the Supplementary Materials section. All of the outcome variables were highly correlated. The results of paired samples *t*-test are shown in Table 2.

**Table 2:** Paired Samples t-Tests in Study 1 (N = 359)

		Paired Differences								
					95% CI					
Pair		*M*	*SD*	*SE*	Lower	Upper	*t*(358)	*p*	*r*	*d*
Willingness for Intergroup Contact										
Pair 1	Chechens - Belarusians	-1.67	2.14	0.11	-1.89	-1.45	-14.79	< .001	.32	0.91
Pair 2	Chechens - Uzbeks	0.19	1.19	0.06	0.06	0.31	2.95	.003	.87	0.08
Pair 3	Chechens - Chinese	-0.07	1.52	0.08	-0.23	0.09	-0.86	.389	.76	0.03
Pair 4	Belarusians - Uzbeks	1.85	2.25	0.12	1.62	2.09	15.61	< .001	.33	0.96
Pair 5	Belarusians - Chinese	1.60	2.04	0.11	1.39	1.81	14.89	< .001	.35	0.90
Pair 6	Uzbeks - Chinese	-0.25	1.34	0.07	-0.39	-0.12	-3.59	< .001	.83	0.11
Endorsement of Discrimination										
Pair 1	Chechens - Belarusians	1.21	1.69	0.09	1.04	1.39	13.57	< .001	.45	0.75
Pair 2	Chechens - Uzbeks	-0.35	1.21	0.06	-0.47	-0.22	-5.43	< .001	.83	0.17
Pair 3	Chechens - Chinese	-0.35	1.50	0.08	-0.51	-0.19	-4.42	< .001	.72	0.18
Pair 4	Belarusians - Uzbeks	-1.56	1.97	0.10	-1.77	-1.36	-15.04	< .001	.43	0.85
Pair 5	Belarusians - Chinese	-1.56	1.99	0.11	-1.77	-1.36	-14.88	< .001	.36	0.89
Pair 6	Uzbeks - Chinese	0.01	1.32	0.07	-0.14	0.13	-0.04	.968	.81	0.01

We found a significant difference with a large effect size for Belarusians, indicating the smallest endorsement of discrimination and the largest willingness for intergroup contact with them, relative to the other groups.

#### Structural Model

The estimated path coefficients of the SEM models are shown in Table 3.

**Table 3:** Model Results for Structural Equation Model: Unstandardized Coefficient (Est.), Standard Error (SE), 95% Confidence Interval (CI), Standardized Coefficient (Std.), z-Value (z) and Significance in Study 1 (N = 359)

			95% CI				
Predictor	Est.	*SE*	Lower	Upper	*z*	*p*	Std.
**Model 1^a^**							
**Generalized Prejudice** (*R^2^* = .35)							
Assimilation	0.203	0.040	0.125	0.281	5.112	< .001	.24
Colorblindness	-0.218	0.034	-0.284	-0.153	-6.511	< .001	-.31
Multiculturalism	-0.032	0.064	-0.159	0.094	-0.504	.615	-.02
Polyculturalism	-0.320	0.059	-0.436	-0.204	-5.418	< .001	-.27
Positive interethnic contact experience	-0.416	0.103	-0.618	-0.213	-4.026	< .001	-.24
**Model 2^b^**							
**Bias against Chechens** (*R^2^* = .35)							
Assimilation	0.204	0.042	0.121	0.287	4.802	< .001	.23
Colorblindness	-0.231	0.036	-0.301	-0.161	-6.464	< .001	-.31
Multiculturalism	-0.046	0.070	-0.182	0.090	-0.663	.507	-.03
Polyculturalism	-0.352	0.063	-0.476	-0.228	-5.557	< .001	-.28
Positive interethnic contact experience	-0.434	0.110	-0.650	-0.218	-3.941	< .001	-.24
**Bias against Belarusians** (*R^2^* = .12)							
Assimilation	0.036	0.029	-0.022	0.093	1.212	.226	.07
Colorblindness	-0.005	0.024	-0.053	0.043	-0.197	.844	-.01
Multiculturalism	-0.136	0.049	-0.231	-0.040	-2.778	.005	-.18
Polyculturalism	-0.046	0.044	-0.132	0.039	-1.064	.287	-.07
Positive interethnic contact experience	-0.235	0.075	-0.383	-0.087	-3.109	.002	-.24
**Bias against Uzbeks** (*R^2^* = .33)							
Assimilation	0.240	0.047	0.148	0.333	5.098	< .001	.24
Colorblindness	-0.244	0.040	-0.322	-0.166	-6.155	< .001	-.30
Multiculturalism	-0.024	0.077	-0.174	0.126	-0.312	.755	-.02
Polyculturalism	-0.370	0.070	-0.507	-0.232	-5.271	< .001	-.27
Positive interethnic contact experience	-0.461	0.121	-0.699	-0.224	-3.808	< .001	-.22
**Bias against Chinese** (*R^2^* = .29)							
Assimilation	0.187	0.046	0.096	0.278	4.023	< .001	.20
Colorblindness	-0.241	0.039	-0.318	-0.164	-6.102	< .001	-.31
Multiculturalism	-0.042	0.076	-0.191	0.106	-0.556	.578	-.03
Polyculturalism	-0.283	0.069	-0.419	-0.148	-4.099	< .001	-.22
Positive interethnic contact experience	-0.441	0.120	-0.675	-0.206	-3.683	< .001	-.23

^a^Global model fit: χ^2^ (83, *N* = 359) = 175.82, *p* < .001; CFI = .970; RMSEA [90% CI] = .056 [.044, .067]; SRMR = .053. ^b^Global model fit: χ^2^(66, *N* = 359) = 148.03, *p* < .001; CFI = .974; RMSEA [90% CI] = .059 [.046, .072]; SRMR = .047.

The initial structural model (explaining generalized intergroup bias) had a poor fit: χ^2^(89, *N* = 359) = 527.82, *p* < .001; CFI = .860; RMSEA [90% CI] = .117 [.108, .127]; SRMR = .056. We turned to the modification indices adding residual correlations between the variables assessing endorsement of discrimination given the high correlations between them (ranging from .34 to .72, the average value was .50); after these modifications, Structural Model 1 had an acceptable global fit: χ^2^(83, *N* = 359) = 175.82, *p* < .001; CFI = .970; RMSEA [90% CI] = .056 [.044, .067]; SRMR = .053. The explained variation of generalized intergroup bias was 35%. The relation between multiculturalism and generalized intergroup bias was non-significant; generalized intergroup bias was negatively correlated with positive interethnic contact experience, colorblindness, and polyculturalism, and positively correlated with assimilation; all other relationships in the model were also significant. The explained variation of intergroup bias against Belarusians by generalized prejudice as the second-order latent factor was only 22%; in contrast, it ranged from 82% to 95% for other groups.

The second structural model (explaining specific intergroup bias) had an acceptable global fit: χ^2^(66, *N* = 359) = 148.03, *p* < .001; CFI = .974; RMSEA [90% CI] = .059 [.046, .072]; SRMR = .047. The explained variation of specific intergroup bias ranged from 12% to 35%. A common pattern for intergroup bias against Chechens, Uzbeks, and Chinese was observed; namely there were negative associations with positive interethnic contact experience, colorblindness, and polyculturalism and positive associations with assimilation. However, intergroup bias against Belarusians was negatively associated only with positive interethnic contact experience and multiculturalism. Taking into account the positive correlation between multiculturalism and polyculturalism (*r* = .32, *p* < .001), if we constrained the path from multiculturalism to bias against Belarusians to be zero in Model 2, then the path from polyculturalism to bias against Belarusians becomes significant (β = -.13, *p* = .036). The resulting SEM model 1 (explaining generalized intergroup bias) is shown in Figure 1.

### Discussion

In this first study, we tested the structural model of the relationships between assimilation, colorblindness, multiculturalism, polyculturalism and generalized and specific intergroup bias, where generalized bias was considered a common second-order latent factor composing intergroup bias against Chechens, Belarusians, Uzbeks, and Chinese. The results showed that only colorblind and polycultural diversity ideologies, taking into account the positive interethnic contact experience, demonstrated negative associations with generalized and specific intergroup bias against Chechens, Uzbeks, and Chinese but not with intergroup bias against Belarusians, which was negatively associated with only multiculturalism (see Table 3). The differences might indicate the lack of an outgroup homogeneity effect and accentuation of various attitudes towards ‘valued’ ethnic groups whose language and culture is similar to Russians and ‘devalued’ ethnic groups against whom Russians already have negative cultural stereotypes or whose culture and religion may be felt to differ considerably from their own (Montreuil & Bourhis, 2001). This potential preference for Belarusians is also clearly illustrated by comparisons in the levels of endorsement of discrimination and willingness for intergroup contact (Table 2), and the difference in factor loadings by the common outcome variables in our results (Figure 1). A similar pattern was observed by Bessudnov (2016): Ukrainians and Moldovans were more acceptable to Russians than immigrants from the Caucasus and Central Asia due to perceived cultural differences and ethnic hierarchy based on stereotypes. Immigrants from Central Asia and the Caucasus are perceived as unskilled manual laborers and perceived as a low-status group (Grigoryev et al., 2019; see also Lee & Fiske, 2006; Linssen & Hagendoorn, 1994). Moreover, according to the ingroup projection model, the status of particular ethnic groups depends on the degree of similarity with prototypical acceptable groups and is usually based on cultural and religious similarity (Minescu & Poppe, 2011; see also Grigoryan, 2020; Grigoryev et al., 2019). Assimilation showed a clearer negative pattern, being associated with poor relations and negative attitudes, which was consistent with the results obtained from recent research (e.g., Whitley & Webster, 2019).

Finally, we discovered that our social ecological/contextual variables corroborated the well-established finding by Pettigrew and Tropp (2006) that, in general, positive contact experience can reduce intergroup bias as it moderates cultural differences and the ethnic hierarchy.

## Study 2

In our second study we attempted to reproduce and extend on the findings of Study 1 by adding implicit bias as the main outcome variable, as explicit measures may not adequately capture bias.

### Method

#### Participants

The total sample comprised 117 ethnic Russian students of HSE University in Moscow. It included women (61.5%) and men (38.5%), aged from 17 to 43 years (*M* = 22.1, *SD* = 6.7).

#### Procedure

The data were collected online in 2018 via email invitations of students. Otherwise, the procedure was the same as in Study 1. IAT was applied to measure implicit bias against target ethnic groups. Participants were presented with a set of visual stimuli (pictures associated with particular ethnic group) to sort quickly to one of two categories associated with pleasant (e.g., “joy”) or unpleasant feelings (e.g., “disgust”). There were seven stimuli for each of the three ethnic groups (Chechens, Belarusians, and Chinese) in 21 sessions.

#### Measures

##### Antecedent Variables

**Diversity Ideologies.** The measures were the same as in Study 1: assimilation, α = .81; colorblindness, α = .75; multiculturalism, α = .73; polyculturalism, α = .82.

##### Covariate Variables

**Behavioral Experience.** We assessed behavioral experience with Chechens, Belarusians and Chinese with four items (Boyle et al., 2014). Positive behavior (two items): “How often did you help Chechens?”. Negative behavior (two items): “How often did you come into conflict with Chechens?”. We used 5-point Likert scale (from 1 = *never* to 5 = *very often*), α > .42 for all considered groups.
**Perceived Similarity.** We used 100-degree thermometer scale (from 0 to 50 = *not similar*; 50 = *do not know*; from 60 to 100 = *very similar*) to assess the perceived degree of similarity with target ethnic groups (Boyle et al., 2014): “How similar are these ethnic groups to your own ethnic group?”

##### Outcome Variables

**Explicit Bias.** We used 100-degree thermometer scale (from 0 to 50 = *not warm*; 50 = *do not know*; from 60 to 100 = *very warm*) to assess perceived degree of warmth of target ethnic groups (Boyle et al., 2014): “How do you feel about these ethnic groups?” Lower thermometer values represent higher levels of explicit bias.
**Implicit Bias.** We used the implicit association test (IAT) with computing D-scores (according to Greenwald et al., 2003). Greater D-scores represent higher levels of implicit bias.

#### Data Analysis

We followed the main steps by Study 1 and used bivariate and partial correlation analysis and regression analysis. We abandoned the SEM approach because the model would have a large number of parameters with a modest sample size, which would not provide convergence and stable estimates. In addition, we believed that we had fully addressed our research questions using these methods. Hence, SEM models would be redundant.

### Results

The results of correlation analysis are shown in Table 4.

**Table 4:** Bivariate and Partial Correlations between Diversity Ideologies and Intergroup Bias in Study 2 (N = 117)

Variable	Assimilation	Colorblindness	Multiculturalism	Polyculturalism
Chechens				
*Covariate*				
Positive behavior	.16/.24**	.13/.25**	-.04/.07	-.04/-.08
Negative behavior	.12/.03	-.27**/-.29***	.01/-.15	.04/.08
Similarity	-.15/-.06	.21*/.03	-.10/-.11	.05/.14
*Outcome*				
Thermometer	-.17/-.11	.16/-.10	-.09/-.07	-.04/-.03
D-score	-.08/-.03	-.08/.04	.09/.20*	-.06/-.14
Belarusians				
*Covariate*				
Positive behavior	.06/-.29***	-.12/.05	.10/.09	.08/-.03
Negative behavior	.08/.17	-.09/-.09	-.17/-.16	-.11/-.05
Similarity	.06/-.04	-.05/.09	.16/.10	.14/-.03
*Outcome*				
Thermometer	.20*/.33***	-.13/-.20*	.17/-.06	.17/.07
D-score	-.03/.01	-.04/-.14	.05/.02	.01/.02
Chinese				
*Covariate*				
Positive behavior	-.18*/-.14	-.21*/-.30***	.11/-.02	.16/.14
Negative behavior	.13/.02	-.01/.22*	.05/.14	.01/-.07
Similarity	-.08/.04	.28**/.22*	-.04/.04	.05/-.05
*Outcome*				
Thermometer	-.18/-.18	.03/.17	.03/.04	.13/.06
D-score	-.03/-.08	-.05/-.14	-.13/-.14	-.19*/-.05

*p < .05. **p < .01. ***p < .001.

The results of the regression analysis are available in Appendix B available in the Supplementary Materials section. These results indicated that assimilation was positively related to explicit bias against Chechens and Chinese and negatively with explicit bias against Belarusians. The results of partial correlations showed that assimilation was also negatively associated and colorblindness was positively associated with explicit bias against Belarusians, whereas multiculturalism was positively associated with implicit bias against Chechens. In general, more proximal variables (positive or negative contact experience and perceived group similarity) were more robust predictors of intergroup bias than diversity ideologies.

### Discussion

The results indicated that negative implicit bias towards Chechens was associated with emphasizing cultural differences with this group (multiculturalism). In contrast, ignoring such differences (colorblindness) was associated with explicit bias against Belarusians but reducing such differences (assimilation) was associated with positive attitudes towards them. However, in one case reducing differences were also associated with explicit bias against Chinese. These patterns echo Brewer's optimal distinctiveness (Brewer, 2003): (1) if the groups are similar, focusing on differences will lead to positive attitudes, because it reduces the distinctiveness threat; (2) if the groups are very different, emphasizing differences makes them seem even more different and the optimal distinctiveness is not achieved.

## Study 3

In Study 3, we examined relations between diversity ideologies and specific forms of intergroup bias. Our rationale was to examine if the pattern of associations was similar to more generic biases.

### Method

#### Participants

The total sample of 225 participants included 41.8% women and 58.2% men, aged 16 to 82 (*M* = 31.2, *SD* = 12.7); 57.8% reported a university education, 40.0% identified as Russian Orthodox Christians, and 28.0% as students.

#### Procedure

The same as Study 1. The data were collected in 2018.

#### Measures

All measures used a 7-point Likert scale. Measures were scored such that higher scores indicated stronger endorsement of the concept.

##### Antecedent Variables

**Diversity Ideologies.** The measures were the same as in Study 1: assimilation, α = .78; colorblindness, α = .89; multiculturalism, α = .77; polyculturalism, α = .83.

##### Covariate Variables

**Ethnic Identification.** We used two items to assess ethnic identification of our participants (Verkuyten & Yildiz, 2007, translated by Grigoryev & Berry, 2017), e.g., “I consider myself a Russian” (α = .89).
**National Identification.** We used two items to assess national identification of our participants (Verkuyten & Yildiz, 2007, translated by Grigoryev & Berry, 2017), e.g., “I consider myself as a citizen of Russia” (α = .85).

##### Outcome Variables

**Antisemitism**. We used five items to assess bias against Jews (Padovan & Alietti, 2012), e.g.: “Jews have too much power in the business world and in international financial markets” (α = .91).
**Fear of Muslims.** We used sixteen items to assess bias against Muslims (Lee et al., 2009), e.g.: “If possible, I would avoid going to places where Muslims would be” (α = .97).

#### Data Analysis

We followed the main steps by Study 1 and used bivariate and partial correlation analysis and regression analysis.

### Results

The correlation matrix is shown in Table 5, while the regression analysis is available in Appendix C available in the Supplementary Materials section.

**Table 5:** Bivariate and Partial Correlations Between Diversity Ideologies and Intergroup Bias in Study 3 (N = 225)

Variable	Assimilation	Colorblindness	Multiculturalism	Polyculturalism
*Covariate*				
Ethnic Identification	.04/.08	-.21**/-.25***	.22**/.11	.05/-.05
National Identification	-.08/-.05	.07/.17**	.13*/.05	.09/.01
*Outcome*				
Antisemitism	.13*/.02	-.38**/-.26***	.24**/.15*	.04/.05
Fear of Muslims	.32**/.31***	-.28**/-.13*	.02/.08	-.25**/-.28***

*p < .05. **p < .01. ***p < .001.

The results indicated that assimilation and multiculturalism had positive associations with specific bias while colorblindness and polyculturalism had negative associations with it.

### Discussion

Largely replicating the findings of Study 1, we found the four ideologies to be a stronger predictor of intergroup attitudes when the target group is more culturally distinct. Together, the four ideologies explained 23% of variance in fear of Muslims, but only 18% of the variance in Antisemitism. As before, the positive link of assimilation with intergroup bias was significant for the culturally distinct group of Muslims, but not significant for the relatively nonvisible and more assimilated group of Jews. However, Jews are not as culturally close to and accepted by the Russian majority as Belarusians in Study 1. Whereas in the case of Belarusians only multiculturalism was associated with more positive attitudes towards them, in the case of Jews the results showed that multiculturalism was associated with more negative attitudes.

The positive role of colorblindness in intergroup attitudes was replicated both for attitudes towards Muslims and towards Jews. We found a negative link between polyculturalism (thinking about interconnectivity) and for fear of Muslims, but not for Antisemitism (note that we also did not find this relationship for the culturally similar Belarusians in Study 1). Also, in parallel with Study 1, multiculturalism was associated with intergroup attitudes only in the case of Antisemitism, but not fear of Muslims. However, the direction of this association was reversed: the more participants endorsed multiculturalism, the more Antisemitism they expressed. This surprising finding might reflect some unique position that the Jewish minority holds in Russian society.

Overall, these findings once again suggest that the relationships of diversity ideologies and intergroup attitudes strongly depend on the specific outgroup under consideration. The positive role of colorblindness and polyculturalism and the negative role of assimilation in intergroup attitudes of Russians seem to be robust, although the effects are often weaker for culturally similar groups. However, we found no relationship between the ideology of multiculturalism (emphasizing differences) and outgroup attitudes for the majority of target groups, and the two target groups that are exceptions to this rule (i.e., Jews and Belarusians), show different directions of the effect.

## General Discussion

In the current series of studies we examined diversity ideologies and noted differences between measures of explicit versus implicit bias: only colorblindness and polyculturalism had negative associations with *explicit* bias against all groups except against Belarusians, which was only negatively associated with multiculturalism, possibly because of the salient categorization activates positive cultural stereotypes. Emphasizing differences (multiculturalism) with Chechens was associated with *implicit* bias. Encouraging an emphasis on differences in some contexts may not always lead to the intended effects such as intergroup harmony or positive interethnic attitudes, and in some cases, may possibly even backfire. Relatedly, other research has shown that priming multiculturalism can be associated with greater prejudice in those who identify with their ethnicity (Morrison et al., 2010).

Stereotypes can trigger a categorization process (or vice versa) and generate an ethnic hierarchy on the basis of the number and intensity of negatively evaluated differences (Hagendoorn, 1993). Multiculturalism is characterized by more pronounced positive and negative stereotypes towards outgroups (Wolsko et al., 2000). Thus, we supposed that the cultural closeness and positive cultural stereotypes towards Belarusians, which are prevalent among Russians (see Grigoryev et al., 2019), results in less intergroup bias against them. Additionally, emphasizing the differences is not a threat to the mainstream cultural group in this case, since the differences are superficial and do not impact on norms, values, and beliefs. It is far easier to support cultural pluralism when it comes to close groups (i.e., Belarusians), in contrast to culturally more distant groups (i.e., Chechens, Uzbeks, and Chinese). Disparities in norms, values, and beliefs which may threaten the cultural security of the mainstream group may lead to a preference for other ideological approaches towards dealing with immigrants than multiculturalism (Grigoryev & van de Vijver, 2018)^5^5Cultural policy is facing similar difficulties in Europe (i.e., very distant cultural groups having difficulty with integration). Western Europeans had relatively positive/neutral experiences with Eastern Europeans (culturally close), but this over generalization of policy to other groups is arguably creating a great deal of tension between segments of the native born European population and immigrants from the Middle East and Africa (Murray, 2017).. Hence, assimilation may be the reactionary response to perceived threats from culturally distant groups (Grigoryev et al., 2018b, 2020), a strategy which restores the cultural security and preservation of the historically dominant group by imposing the mainstream culture on immigrants (Guimond et al., 2013).

Moreover, in the case of Belarusians (Study 1), multiculturalism (emphasizing differences) completely shared the variation of polyculturalism (thinking about interconnectivity) and additionally explained the unique variation of the outcome variables; this finding is similar to the results for endorsement of the celebration of *Harmony Day* in Australia obtained by Pedersen et al. (2015). That is, polyculturalism had the beneficial effect for all considered outcomes. Apparently, it is indeed possible to improve intergroup relations by recognizing the connections among groups through their shared past and current interactions and exchanges (Rosenthal & Levy, 2012) that really took place for the considered groups. Yet again, the lack of significant relationships between colorblindness and intergroup bias against Belarusians, is in line with the reasoning of the previous paragraph, due to the features of the intergroup relations with this ethnic group in Russia and intergroup differentiation. Instead, for other groups, colorblindness may be a reflection of the famous cliché ‘no bad nations, but bad people’, which is quite common in public discourse in Russia. Thus, colorblindness may help to partially reduce judging individuals based on ethnic categories, which are vulnerable to outgroup-based prejudice. Colorblindness looks like a form of French republicanism (secularism, universalism) and European model of the national state on a civic basis (Guimond et al., 2014). However, colorblindness also has some limitations. For example, cognitive studies of the automatism of the categorization process cast doubt on the possibility of avoiding categorization: after some time, a person automatically places an object in a certain category (see Correll et al., 2008). Others maintain that ignoring race may ignore the existence of racial discrimination, i.e., it is not difficult to move from an egalitarian to an anti-egalitarian understanding of colorblindness depending on political motives and current events (Apfelbaum et al., 2012; Guimond et al., 2013). A recent study in New Zealand demonstrated the mechanism of the functioning of such an anti-egalitarian understanding of colorblindness (Yogeeswaran et al., 2018). However, diversity ideologies can be very sensitive to the country context (Guimond et al., 2014); therefore, the results obtained in other countries should be compared with great caution.

### Limitations

Our study considered ideologies that included only the maintenance of cultural diversity aspect, and not the participation and inclusion aspect. Three other ideologies of multicultural ideology (Berry & Kalin, 1995); interculturalism (Gagnon & Iacovino, 2016; Scott & Safdar, 2017; Verkuyten et al., 2020); and omniculturalism (Moghaddam, 2012) include not only cultural diversity but also the social participation and inclusion of diverse groups in the life of the larger national society (Grigoryev & Berry, 2021). Future research should examine in a comparative perspective the role of these three ideologies in interethnic bias.

The concept of mutuality is essential for understanding intercultural relations (e.g., Berry, 2017; Berry et al., 2022). Does bias appear in the views of the dominant and non-dominant groups towards each other? However, in the present studies, we examined diversity ideologies only from the point of view of the majority cultural group, even though there are differences for majority and minority groups (e.g., Ryan et al., 2007).

Finally, while we addressed some social contextual variables, including ethnic density, their moderating and mediating effects need to be further investigated (Jurcik et al., 2015; Visintin et al., 2017b). Variables such as intergroup threat (e.g., Morrison et al., 2010; Scott & Safdar, 2017) and authoritarianism (e.g., Levin et al., 2012; Perry et al., 2015; Yogeeswaran et al., 2017) may also more finely tune the obtained relationships. Although a low internal consistency of multiculturalism (in Study 1) relative to other measures of diversity ideologies did not pose serious threats to our results, further research using a more reliable measure is necessary because a high error variance attenuates effects.

### Further Research

Further research is needed in different contexts from a comparative perspective, which will help to establish under what conditions these principles of diversity ideologies (or their combination) are most effective in achieving mutual acceptance. In other words, the following questions are still open: (1) In which cases is it better for people to perceive each other as separate individuals, and not as members of groups? (2) Under what circumstances would social categorization play a role in attitude formation (since a positive/negative attitude towards one group member can be transferred to other members of this group)? (3) In which situations is it better to attach importance to how cultural groups have interacted, influenced and mutually enriched each other throughout history (and continue to do so today), viewing members of all racial and ethnic groups as deeply interconnected? (4) What is the optimal distinctiveness for specific groups within their respective contexts? Other research may also explore the evolutionary nature of cognitive biases, which some have argued, may have been ancestrally adaptive from a fitness perspective, even if they may not appear logical (see Haselton et al., 2015). Thus, it is possible that under certain conditions of threat or biological state, certain diversity ideologies may be favored over others, indicative of dynamic processes.

## Conclusion

Our findings from the Russian context help shed light on the functioning of diversity ideologies in the aspects of maintenance of cultural diversity, and in the difference in the cultural similarity of the dominant group towards ethnic minority outgroups. Assimilation primarily involves the motivation to unite society into one single united group and the rejection of non-mainstream cultures, and represents only one approach to adapting to a society especially for culturally distant groups. Seemingly, multiculturalism, which emphasizes differences, is useful only if there are positive intergroup stereotypes; in the case of negative cultural stereotypes, however, this may invoke a negative bias. Although replication and further experimental research is needed, our study serves as a reminder of how emphasizing differences may inadvertently be associated with more negative attitudes (see Morrison et al., 2010), especially towards more culturally distant groups. It also highlights how implicit and explicit bias may manifest slightly differently.

Instead, both colorblindness and polyculturalism were generally associated with less negative bias in Russia. Colorblindness appeals to a common citizen identity by eliminating ethnic differences and complicating ethnic categorization and judgments. Polyculturalism emphasizes positive historical experiences of intercultural relations and probably corresponds to available rooted views, such as Soviet internationalism and the hope for solidarity between ethnic groups in Russia. Whether this hope was ever achieved historically is debatable, but our study highlights how context and local worldviews need to be carefully considered when studying diversity ideologies. Our findings indicate a great need to take into account the principles of Brewer’s optimal distinctiveness theory (Brewer, 2003) for intercultural relations. Thus, our findings may be suggestive of a compromise strategy needed to maintain some 'optimal' level of perceived cultural difference in intercultural relations—favoring more similarity if different, and more difference if similar.

## Supplementary Materials

For this article the following Supplementary Materials are available via PsychArchives (for access see the Index of Supplementary Materials below)

- Three appendices with the descriptive statistics of each study, including means, standard deviations, partial correlations, and regression analyses

BatkhinaA.
BerryJ. W.
JurcikT.
DubrovD.
GrigoryevD.
 (2022). Supplementary materials to "More similarity if different, more difference if similar: Assimilation, colorblindness, multiculturalism, polyculturalism, and generalized and specific negative intergroup bias"
[Appendices]. PsychOpen. 10.23668/psycharchives.12170
PMC978073636605093

## Data Availability

Data is freely available at Supplementary Materials

## References

[sp1_r1] Batkhina A. Berry J. W. Jurcik T. Dubrov D. Grigoryev D. (2022). Supplementary materials to "More similarity if different, more difference if similar: Assimilation, colorblindness, multiculturalism, polyculturalism, and generalized and specific negative intergroup bias" [Appendices]. PsychOpen. 10.23668/psycharchives.12170 PMC9780736 36605093

